# Novel Surface-Modified Bilosomes as Functional and Biocompatible Nanocarriers of Hybrid Compounds

**DOI:** 10.3390/nano10122472

**Published:** 2020-12-10

**Authors:** Ewelina Waglewska, Agata Pucek-Kaczmarek, Urszula Bazylińska

**Affiliations:** Department of Physical and Quantum Chemistry, Faculty of Chemistry, Wroclaw University of Science and Technology, Wybrzeze Wyspianskiego 27, 50-370 Wroclaw, Poland; ewelina.waglewska@pwr.edu.pl

**Keywords:** polymeric bilosomes, biosurfactants, nanoparticles, drug delivery, anticancer treatment

## Abstract

In the present contribution, we demonstrate a new approach for functionalization of colloidal nanomaterial consisting of phosphatidylcholine/cholesterol-based vesicular systems modified by FDA-approved biocompatible components, i.e., sodium cholate hydrate acting as a biosurfactant and Pluronic P123—a symmetric triblock copolymer comprising poly(ethylene oxide) (PEO) and poly(propylene oxide) (PPO) blocks Eight novel bilosome formulations were prepared using the thin-film hydration method followed by sonication and extrusion in combination with homogenization technique. The optimization studies involving the influence of the preparation technique on the nanocarrier size (dynamic light scattering), charge (electrophoretic light scattering), morphology (transmission electron microscopy) and kinetic stability (backscattering profiles) revealed the most promising candidate for the co-loading of model active compounds of various solubility; namely, hydrophilic methylene blue and hydrophobic curcumin. The studies of the hybrid cargo encapsulation efficiency (UV-Vis spectroscopy) exhibited significant potential of the formulated bilosomes in further biomedical and pharmaceutical applications, including drug delivery, anticancer treatment or diagnostics.

## 1. Introduction

In recent years, in the field of nanotechnology, the development of functional carriers by embedding various molecules, e.g., polymers, proteins, polysaccharides or nucleic acids into their structure by using various structural design techniques has been observed [1]. These structuring agents allow the modification of key carrier properties such as increasing stability, ensuring biodegradability and/or reducing toxicity. Thanks to this, the functionalized carrier becomes an intelligent tool for specific purposes, such as treatment or diagnosis of many diseases [2,3,4]. Accordingly, various types of functionalized nanocarriers, involving different structural design processes, have been extensively studied in our research group [5,6,7].

Liposomes are currently some of the most often studied nanocarriers for the delivery of active substances. They are spherical-shaped vesicular structures with a size range of 25 nm to a few microns, formed from phospholipids containing a hydrophilic head and two hydrophobic tails [8,9]. The use of liposomes as a drug delivery system offers numerous advantages such as: high biodegradability and biocompatibility with low toxicity, ability of self-assembly, possibility of simple removal from the body and increased efficacy and bioavailability of encapsulated compounds [10,11]. Most importantly, this type of nanocarrier can ensure encapsulation of both hydrophobic and hydrophilic agents, in the phospholipid bilayer and aqueous core, respectively [12,13]. These vesicular nanostructures also provide the ability to modify key physicochemical properties, i.e., size and surface, which is crucial in the subsequent application [14]. From the 1960s onwards, liposomes have been used in numerous food, cosmetic and agricultural fields and, above all, in the pharmaceutical industry [15]. The development of this type of nanocarrier is very dynamic and promising, as evidenced by the fact that liposomes were the first nanoscopic drug delivery system to be approved for clinical practice (Doxil^®^, in 1995) [16].

The use of liposomes is mainly limited by their insufficient physical (the processes of destabilization such as aggregation, flocculation or coalescence, which ultimately lead to changes in the size of the nanostructure) and chemical (hydrolysis of ester bonds or peroxidation of the unsaturated acyl chain) stability [17]. To enhance the stability and drug loading efficiency of conventional vesicular-type vehicles, novel structural liposomes were developed involving supporting additives such as cholesterol, ethanol or non-ionic surfactants. The incorporation of alcohol may increase lipid fluidity and cell membrane permeability, which leads to the creation of pliable and soft nanovesicles. For this reason, increasing ethanol concentration (to 40–45%) will improve the entrapment efficiency of drugs and other bioactive compounds. Ethanol also provides a negative charge for the surface of modified liposomes, thus avoiding aggregation of these vesicles because of electrostatic repulsion [18]. Another very important function of this alcohol is the “playing of the humectant role” to strengthen the penetration capacity into the deeper skin layers, which in turn increases the efficiency of the delivery of active substances [19]. Niosomes are systems formed from non-ionic surfactants with the addition of cholesterol. Transferosomes are the second group of liposomes composed of phospholipids with the presence of additional single-chain surfactants, also called edge activator. The third group includes ethosomes—vesicular structures containing phospholipids and a relatively high amount of ethanol [20,21].

The latest literature reports delineate the next generation of “soft” lipid vesicular nanocarriers—bilosomes in which the role of edge activator is acted by bile salts such as deoxycholic acid, sodium cholate, deoxycholate, taurocholate, glycocholate or sorbitan tristearate. The addition of these biosurfactants increases the colloidal stability of the system compared to the conventional liposome [22]. Bilosomes can facilitate the absorption of drugs and consequently improve the bioavailability of the encapsulated active cargo [23]. The presence of bile salts improves the susceptibility of the nanocarrier to degradation in the gastrointestinal tract and increases permeability through mucous membranes, which is advantageous in the oral administration of bilosomes [24,25]. In addition, their transdermal delivery is enhanced due to the greater flexibility of the structure, which results in more efficient penetration of the stratum corneum and higher penetration of the skin-deep layers [26,27].

Furthermore, the stability and functionality of any vesicular-type nanocarrier may be enhanced by adding a biocompatible triblock polymeric compound from the Pluronic (so-called Poloxamer) family. Increasing the stability of the hydrophobic surface of colloidal dispersions is possible due to the presence of external hydrophilic poly(ethylene oxide) groups in the Pluronic structures, which form a specific steric barrier. Because of their mildness and low immune response, Pluronics have been approved as safe by the FDA (Food and Drug Administration) and EMA (European Medicines Agency) for pharmaceutical applications as well as additives to food and agricultural products [28,29]. The presence of a Pluronic-type polymer allows reduction of the risk of recognition and uptake by the mononuclear phagocyte system (MPS), thus extending the circulation time of nanostructures in the body. Moreover, nanomaterials based on these copolymers do not cause irritation when applied subcutaneously/topically and they are eliminated in the urine by filtration through the kidneys [28,30].

**Scheme 1:**
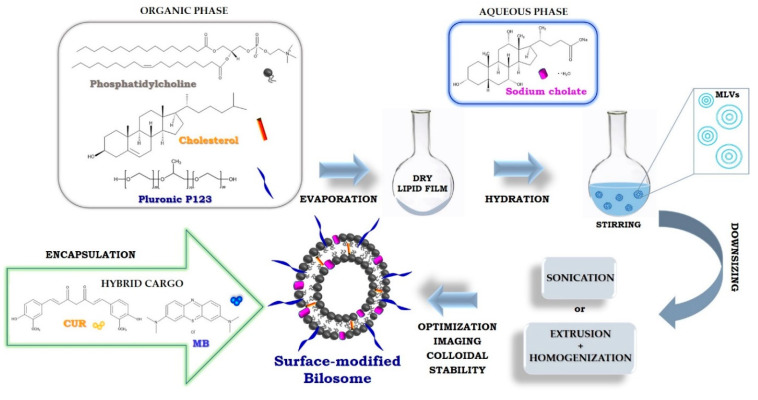
Schematic representation of the fabricated functional bilosome for double encapsulation of the active cargo with the structures of the main components.

In the present contribution (Scheme 1), we have designed and fabricated a phosphatidylcholine/cholesterol-based liposome modified by sodium cholate hydrate. Furthermore, the surface of the novel bilosomes was functionalized by the addition of a biocompatible symmetric triblock copolymer comprising poly(ethylene oxide) (PEO) and poly(propylene oxide) (PPO) units in an alternating linear fashion, PEO-PPO-PEO—Pluronic P123. The influence of various concentrations of the formulation components and the structuring agents and the type of nanocarrier preparation method (thin-film hydration, sonication and extrusion in combination with homogenization technique) was investigated to characterize obtained vesicular structures—their size, morphology and kinetic stability. To the best of our knowledge, this is the first report to compare the physicochemical parameters and kinetic stability of polymer-functionalized bilosomes synthesized by different fabrication methods. Furthermore, this is the first case in which the bilosome structure is modified with Pluronic P123. To test the efficiency of encapsulation in the novel optimized bilosomes, two photosensitizing and anticancer agents: hydrophobic curcumin and hydrophilic methylene blue were applied as a model hybrid cargo.

## 2. Materials and Methods

### 2.1. Materials

L-α-Phosphatidylcholine (egg yolk, ~60%, confirmed by thin layer-chromatography (TLC)) and sodium cholate hydrate (≥97%; dried material) were purchased from Sigma-Aldrich. Cholesterol was provided by CRODA Inc. (East Yorkshire, England). Poly(ethylene glycol)-block-poly(propylene glycol)-block-poly(ethylene glycol) Pluronic^®^ P-123 (average *M*_n_ ~5800) employed as a polymer for nanocarriers stabilization, was acquired from Sigma-Aldrich (Poznań, Poland). Methylene Blue (MB) Injection (1%; Akorn, Inc., Lake Forest, IL, USA) and curcumin (CUR) (Sigma-Aldrich, Poznań, Poland) were used as active model compounds. Chloroform and tetrahydrofuran (THF) was supplied by Avantor Performance Materials Poland S.A. (formerly POCH S.A., Gliwice, Poland). Distilled water was used for all experiments. All the above-mentioned chemicals were of commercial grade and were applied as received.

### 2.2. Preparation of Functional Bilosomes

Bilosomes surface-modified with a triblock copolymer were formulated by the thin-film hydration method at different weights of sodium cholate. The compositions of the various liposomal formulations are presented in Table 1. For stabilization of all nanocarriers, triblock copolymer Pluronic P123 was used in a concentration of 0.6% (see Supplementary Materials). Phosphatidylcholine, cholesterol and Pluronic P123 were dissolved in a round-bottom flask using chloroform. The organic solvent was evaporated under reduced pressure by a rotary evaporator at 40 °C at a speed of 80 rpm. Subsequently, the formed lipid thin film was hydrated using 10 mL of distilled water containing sodium cholate. Glass beads were added to the flask in order to strengthen the detachment of the lipid film. The dispersion was stirred for 2 h on the magnetic stirrer. The resulting suspension of multilamellar vesicles was further downsized to reduce the particle size. A part of the sample was sonicated in the ultrasonic bath for 15 min, while the rest of the liposomal suspension was filtered through a 0.2 µm filter and homogenized using a probe sonicator (Ultrasonic Homogeniser Sonopuls HD 2070.2., Bandelin, Berlin, Germany) in pulse mode for 5 min. Empty bilosomes were stored at a temperature of 4 °C. Double-loaded vesicles were prepared following a similar procedure with the addition of curcumin (CUR) to the lipid phase and methylene blue (MB) to the aqueous phase. In that case, both the active substances with different hydrophilicity were added at a concentration of 0.05 mg/mL.

### 2.3. Characterization Methods

#### 2.3.1. Particle Size and Zeta Potential Analysis

The size distribution of nanocarriers (i.e., the hydrodynamic diameter, D_H_) and polydispersity index (PdI) of the modified liposomes were analyzed by dynamic light scattering (DLS) using a Zetasizer Nano ZS (Malvern Instruments, Worcestershire, UK), with a detection angle of 173° in optically homogeneous square polystyrene cells. Each value was obtained as an average of three subsequent runs of the instrument, with at least 10 measurements. The surface charge (i.e., ζ-potential) of the received vesicles was measured by electrophoretic light scattering (ELS) using a Malvern Zetasizer Nano ZS apparatus. The results are given as an average of three runs, with at least 20 measurements. All the measurements were determined at 25 °C. Each sample was analyzed in three technical replicates and reported in Figures as the mean with standard deviation (SD).

#### 2.3.2. Morphological Characterization Using TEM

The morphology of the obtained bilosomes was studied by transmission electron microscopy (TEM). A drop of the diluted sample was placed on a perforated carbon film-coated copper grid and was left to dry for 1 h before the examination. Then, the samples were observed and imaged using an FEI Tecnai G2 20 X-TWIN (FEI, Hillsboro, OR, USA) transmission electron microscope.

#### 2.3.3. Kinetic Stability by Backscattering Profile

The kinetic stability measurement was carried out for the optimized bilosome formulation. The analysis was performed in a cylindrical glass cell at 25 °C using Turbiscan Lab Expert (Formulaction SA, L’Union, France) by measuring the backscattering (BS) of pulsed near infrared light (λ = 880 nm). BS profiles as a function of sample height were collected and analyzed by the instrument software (Turbisoft version 2.2.0.82). The stability was measured to detect any destabilization phenomena of the polymeric colloidal systems.

#### 2.3.4. Determination of the Bilosome Encapsulation Efficiency

In order to evaluate the encapsulation efficiency of therapeutic compounds in the optimized bilosomal formulation, UV-Vis spectroscopy was applied. Measurements were performed using a Hitachi U-5100 spectrophotometer (Hitachi, Mannheim, Germany) with a 1 cm path length quartz cell. The active cargo content was quantified at *λ*_CUR_ = 426 nm and *λ*_MB_ = 666 nm after the disruption of bilosomes with THF:water (1:1). The substance concentration was calculated using the calibration plot. The percentage of encapsulation efficiency was quantified using the Equation (1): given below.

(1) $$\mathrm{Percentage}\;\mathrm{encapsulation}\;\mathrm{efficiency}\;\left(\mathrm{EE}\%\right)=\;\frac{drug\;content\;in\;bilosomes}{total\;drug\;added}\times \;100\%\;$$

## 3. Results

### 3.1. Physicochemical Characteristics of Bilosomes and Optimization Studies

In our contribution, different types of structured phospholipid vesicles were used as drug delivery systems for potential anticancer therapy to achieve high therapeutic efficiency. Effective accumulation of these nanostructures in target tissues depends on their physicochemical properties such as particle size distribution and surface charge [31]. Thus, developing safe and stable nanoscopic carriers requires the preparation of monodisperse colloidal systems of a specific size. Moreover, the selection of the preparation technique can significantly influence the phospholipid vesicle’s properties. According to the general strategy presented in Scheme 1, the process of structural design of the functional bilosomes involved several stages. In the first step, the dry lipid film was produced by solvent evaporation from the organic phase containing phosphatidylcholine/cholesterol/Pluronic P123 mixture. Next, the formed lipid film was hydrated by using aqueous solution with anionic biosurfactant-sodium cholate, resulting in the formation of multilamellar vesicles (MLVs). However, this kind of nanocarrier may be rapidly captured and removed from blood circulation by the mononuclear phagocyte system (MPS) cells, since their size is over 500 nm [1,26]. The next step was the reduction of MLVs dispersion size by sonication and extrusion in combination with homogenization.

**Figure 1:**
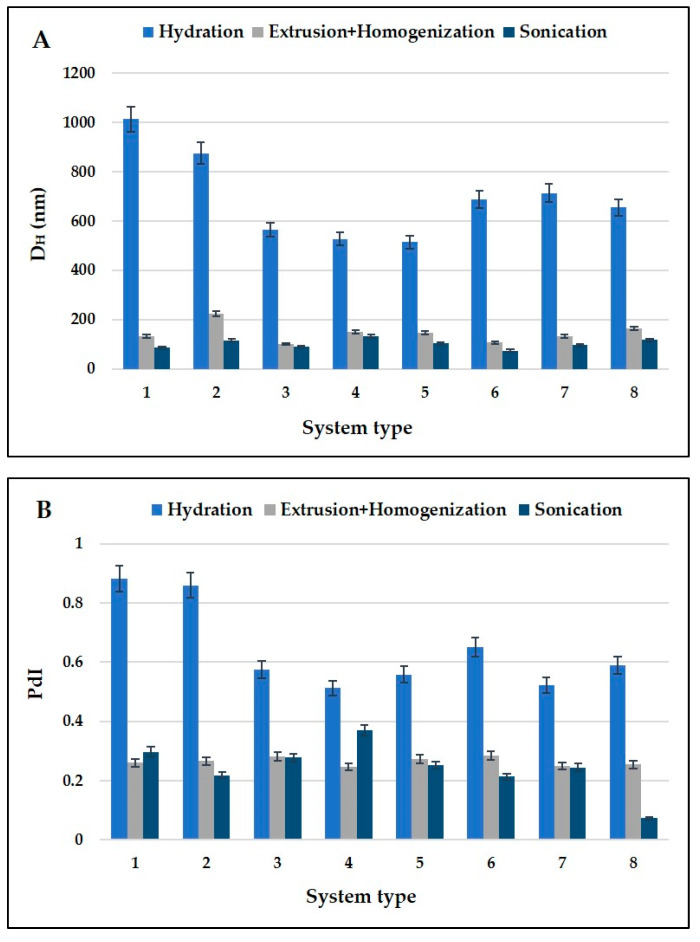
Influence of the modified liposomes preparation methods on the (**A**) vesicle size (D_H_) and (**B**) polydispersity index (PdI).

For the first phase of the process we applied cholesterol molecules, which affect the fluidity of the liposomal bilayer and thus improves the vesicle’s stability [17]. Finally, to enhance nanocarrier stabilization, pharmaceutically-approved non-ionic triblock copolymer (Pluronic P123) was applied. Recent years have shown that Pluronic P123 is one of the most promising poly(ethylene glycol) (PEG)-based amphiphilic block copolymers for controlled and targeted drug delivery. The polymer shell helps to avoid the immune system reaction and consequently extends the blood circulation time of these nanocarriers via the “stealth” effect [32]. In PEO-based polymeric nanostructures, the attached poly(ethylene oxide) chains form a highly hydrated surface of polymer brushes on the surface of vesicles. This effect sterically inhibits hydrophobic and electrostatic interactions between vesicles and plasma proteins, which consequently protects the bilosomes from rapid uptake by the MPS [11]. Significantly, preliminary tests showed that Pluronic P123 exhibits very important biological activity. It restores the sensitivity of the multidrug resistance tumor cells to anticancer agents [30]. Lastly, the possibility of activating the hydroxyl terminal group of PEO-PPO-PEO block copolymer via attachment of new functional groups may contribute to an increase in the efficiency of targeted delivery. This action allows to prevention of interaction of the encapsulated drug with normal cells, which in turn reduces adverse effects of these active substances in the body. The appropriate concentration of polymer was selected according to our preliminary studies (Supplementary Materials Table S1). The effective process of designing our innovative nanostructures required an application of additional lipid bilayer stabilization. Thus, we applied biocompatible bile salt (i.e., sodium cholate), so that our nanostructures could act as more stable carriers than conventional liposomes. Bile salts are biosurfactants, which improve bioavailability of active cargo with absorption limiting factors such as low membrane permeability or poor aqueous solubility [22]. In this context, sodium cholate–Pluronic P123 hybrid nanosystems can contribute to the novel drug delivery systems development (especially for anticancer therapy). Eight types of structured liposomes (systems 1–8 from Table 1) were successfully synthesized in our studies. The averages sizes (hydrodynamic diameters, D_H_) and polydisperity index (PdI) of the obtained nanocarriers were compared depending on their composition and the preparation method used (shown in Figure 1). The control parameters of the polymeric liposomes with various concentrations of Pluronic P123 are summarized in the Supplementary Materials Figure S1.

Concerning the influence of the manufacturing method on the nanostructure size, we observed that the smallest empty nanocarriers with narrow size distribution (PdI < 0.3, except for sample 4) were obtained after sonication, while those only subjected to hydration showed the broadest range of particle sizes and polydispersity (up to 1012 nm and 0.881 PdI, respectively for system 1). In drug delivery applications using phospholipid vesicles, values 0.3 PdI and below are deemed acceptable and indicates a monodisperse population of liposomes [33]. The very low PdI value of sample 8 obtained by sonication technique can be explained by irregular strength of sonic vibrations. In the ultrasonic bath, the sonic signals diverge unevenly, which can have a significant impact on the value of the polydispersity index. Furthermore, the temperature in the bath itself rises, which may also result in different characteristics of the obtained carriers [34]. Furthermore, the colloidal nanoparticles as bilosomes dispersed in the liquid solvent are in continuous motion (called Brownian motion) and are bombarded by particles of the dispersing phase. In a light scattering technique, it is not possible to accurately know how every particle moves in the medium. If the suspended particle is small, the number of colliding particles can be different from variable directions. As a consequence, it may be received at any moment pulses with different intensities, resulting in a difference in the particle size distribution calculated using the Stokes–Einstein equation [35].

Regarding the bile salt influence on the nanocarrier particle size, it should be noted that the majority of the nanosystems stabilized by sodium cholate revealed lower values of hydrodynamic diameter (D_H_) compared to systems 1 and 2. The addition of bile salts into the lipid bilayer affects flexibility of carriers, which consequently leads to decrease in particle size of the modified liposomes. The increase in weight ratio of sodium cholate caused vesicles to enlarge (Figure 1A), which is probably related to the increased medium viscosity because of the higher concentration of bile salt [36].

**Figure 2:**
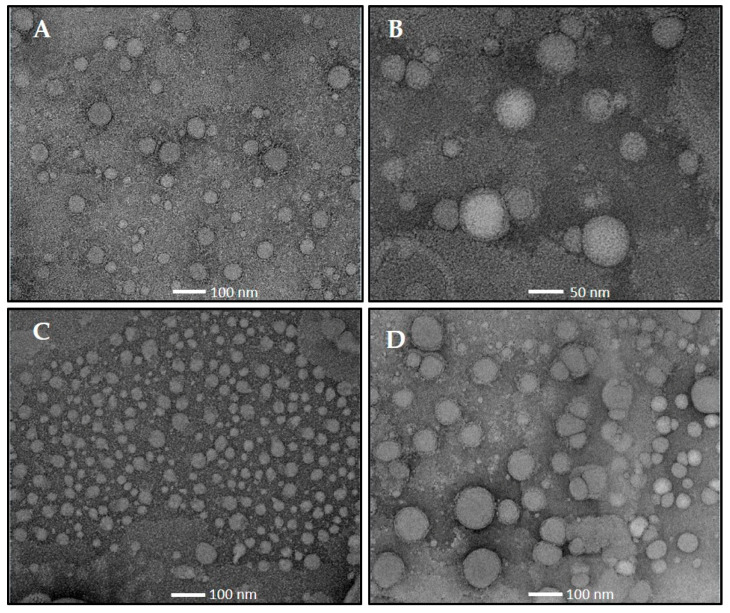
TEM images of (**A**,**B**) bilosomes and (**C**,**D**) the bilosomes surface-modified with Pluronic 123 (respectively, samples 4 and 7 in Table 1).

Transmission electron microscopy (TEM) imaging was performed on bilosomes and surface-modified bilosomes (shown in Figure 2) to reveal the nanocarrier morphological structure. This technique is the most commonly used imaging method for the evaluation of nanomaterial structure. It can rapidly provide information on morphology and size distribution. This is made possible by achieving much-improved resolution, better contour and contrast of images than any other microscope technique [37]. The TEM images revealed the presence of spherical nanostructures with roughly uniform sizes. The addition of polymer did not appear to change the shape and morphology of the obtained nanosystems, but we can observe the monodisperse distribution of functional bilosomes in Figure 2C. Moreover, any enhanced aggregation of the bilosomes was not observed on the found images. Consequently, the imaging data confirmed the results obtained by DLS and showed nanoparticles with sizes of approximately 100 nm.

**Figure 3:**
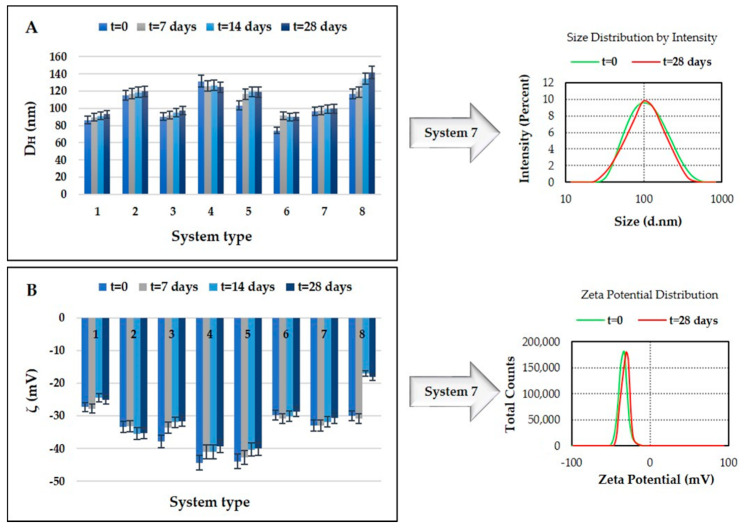
Influence of the storage time on (**A**) mean particle size (D_H_) and (**B**) zeta potential (ζ) for all the samples after sonication process.

**Figure 4:**
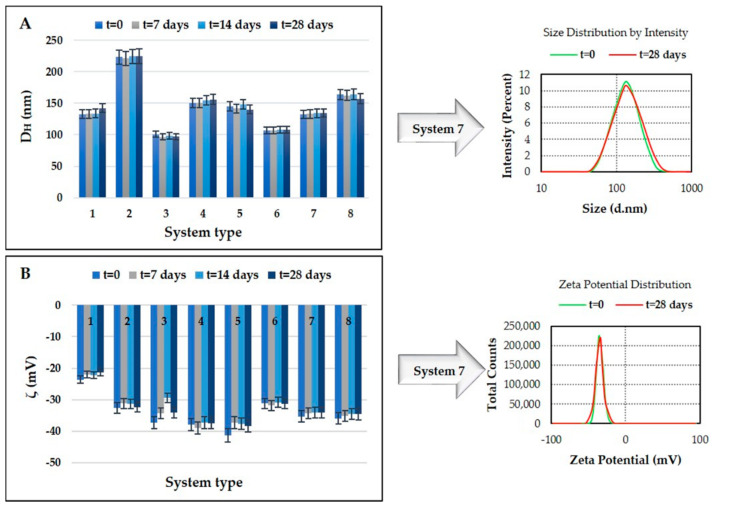
Influence of the storage time on (**A**) mean particle size (D_H_) and (**B**) zeta potential (ζ) for all the samples after extrusion and homogenization processes.

Physicochemical stability is among the most important points in the design of novel nanocarriers intended for drug delivery. Thus, the stability studies of all the bilosomes after sonication (Figure 3) and extrusion in combination with homogenization (Figure 4) after the production process (t = 0 days) and after storage at 4 °C (t = 7 days, t = 14 days and t = 28 days) were performed. Influence of the storage time on mean particle size, ζ-potential and polydispersity index for surface-modified liposomes with various concentrations of polymer is shown in the Supplementary Materials Figure S2.

As it has been presented in Figure 3 and Figure 4, the obtained dimensions and zeta potential values for sample number 7 exhibited minimal changes of those parameters over time in comparison to other samples (maximum increase of about 3 nm for both preparation methods). From Figure 3 and Figure 4, we observed slight changes of D_H_ values of the conventional liposomes (sample 1) after 28 days of storage (increase of hydrodynamic diameter from 86.5 nm to 93.1 nm and from 132.7 nm to 142.4 nm, respectively for sonication and extrusion in combination with homogenization). Moreover, the addition of bile salt influenced the surface charge of the bilosomes in comparison to classic liposomes, shifting the ζ-potential to more negative values, which may be connected to the addition of the anionic biosurfactant. Equally noteworthy was less negative zeta potential for surface-modified bilosomes (systems 6–8) compared to bilosomes without Pluronic P123 (systems 3–5), which might be explained by the addition of polymer. PEG-coated bilosomes show low electrophoretic mobility according to the hydrodynamic resistance given by triblock copolymer [38,39]. The shift in electrical mobility confirmed that post-inserted Pluronic P123 was occupying the surface of our bilosomes. In the case of nanocarriers with the highest sodium cholate concentration (sample 8 after sonication), we observed the poorest sample quality resulting in an increase of the particle size (from 116.7 nm to 141.7 nm) at a simultaneous high increase of the zeta potential value up to −18.2 mV.

These results show that the choice of method affects the size of liposomal formulations. After sonication we obtained smaller nanocarriers with size from 75–120 nm. In contrast, after extrusion in combination with homogenization we received vesicles with size from 100–220 nm. Selection of preparation techniques did not affect the stability of our nanostructures.

After the nanocarriers’ storage time, we selected surface-modified bilosomes (system 7) appropriate for further analysis connected with kinetic stability, which had the most unimodal size (D_H_~100 nm) and the required narrow size distribution (PdI < 0.25). According to the literature data, nanocarriers with size from 100–150 nm may exhibit decreased uptake by mononuclear phagocyte system cells and longer blood circulation times, which is reflected by enhanced accumulation in cancer tissues [33,40]. Furthermore, the pilot stability studies against the nanocarrier agglomeration (time-dependent D_H_ measurements) provided in the standard culture medium, i.e., Dulbecco’s Modified Eagle’s Medium (DMEM) supplemented with 10% fetal bovine serum and antibiotics (for details see Supplementary Materials Figure S3) also suggest biological stability of the obtained bilosomes. For this reason, we decided to choose formulation after sonication for the next stage of our investigation, since its size was optimal for double encapsulation of active cargo, which made these nanocarriers a good candidate for further anticancer therapy.

### 3.2. Kinetic Stability Evaluation of the Optimized Nanocarriers by Backscattering Profiles

The stability of liposomal formulations is the main limiting factor for active substances delivery using this nanosystem. The adverse phenomena occurring during storage time may affect the kinetic stability of liposomal formulations. Liposomes might be subject to aggregation, flocculation and coalescence, which can definitively change vesicle size and also lead to the leakage of the encapsulated drugs. The main reasons for liposomal aggregation are interactions and forces between colloidal nanostructures in suspensions (e.g., short-range Van der Waals interactions). For the prevention of this destabilization process, steric or electrostatic stabilization should be introduced [14,17]. Thus, the stability evaluation of any delivery system is a key issue in designing functional bilosomes.

**Figure 5:**
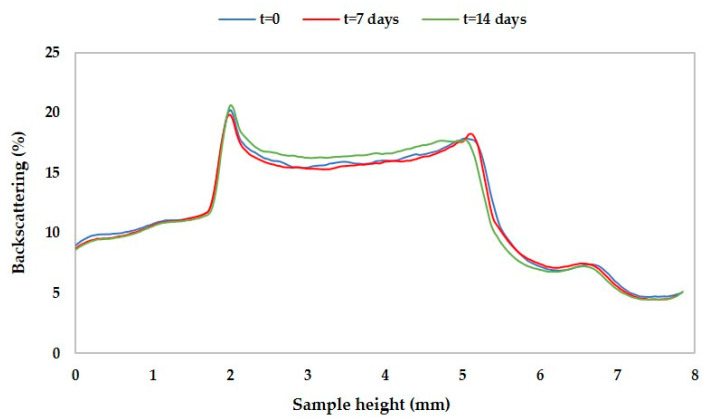
Exemplary backscattering (%) profiles of the obtained functionalized bilosomes as a function of sample height (mm) analyzed over 14 days of storage. Blue line represents measurement at 0 day (freshly prepared bilosomes); red line was recorded after 7 days of their storage, while green line indicates data obtained after 14 days of storage. See description of the system in Table 1.

The kinetic stability of the optimized bilosomes was evaluated by a Turbiscan Lab Expert optical analyzer, which (based on multiple light scattering technologies) enables detection of different types of formulation instability (such as flocculation, sedimentation, coalescence or creaming). Therefore, the turbidimetric method was used to evaluate in detail the backscattering profiles of the functional bilosome formulations (Figure 5). The dynamics of the processes occurring in the formulation under investigation were determined at 0 day (freshly prepared bilosomes) and after 7 and 14 days of storage at 4 °C. A representative Turbiscan plot is shown in Figure 5 for the selected and optimized sample 7. The levels of the backscattering (expressed in percentage) are marked on the ordinate axis, while the height (expressed in mm) is indicated on the abscissa axis. The x-axis corresponds to different levels of the colloidal sample in the measurement vial. From the plot presented in Figure 5, we did not observe significant changes after 14 days of storage time, which indicates no evident particle growth or migration in the nanoparticle dispersion. Typically, rapid destabilization phenomena are characterized by a large separation between the curves, while an overlap of the individual curves indicates slow rate of the destabilization process and consequently very high stability of the analyzed formulation [5,41]. Comparing the formulation stability expressed by backscattering profiles with dynamic light scattering (measurements of nanoparticle size, D_H_ and polydispersity index, PdI), we can observe only a very small increase in the nanocarrier’s size (from 96.5 nm to 98.8 nm). The nanosystem 7 is characterized by narrow size distribution (PdI~0.245) and its low polydispersity is almost unchanged after 14 days of storage (PdI~0.250). The obtained results showed that our functionalized bilosomes were highly stable, and consequently the optimized system 7 was taken for the next step of our contribution involving encapsulation of hybrid model compounds.

### 3.3. Application Potential of the Colloidal Stable Bilosomes in the Hybrid Cargo Encapsulation

**Figure 6:**
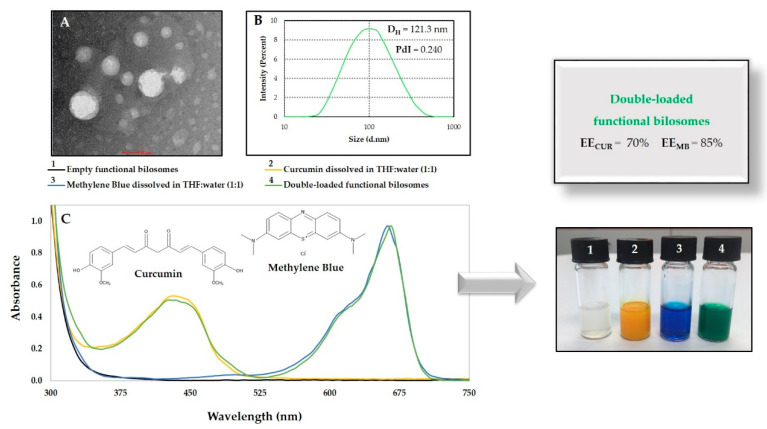
Characteristics of the functional bilosomes after the hybrid cargo encapsulation using (**A**) TEM, (**B**) dynamic light scattering (DLS), (**C**) UV-Vis spectra. UV-Vis absorption spectra and images of the (**1**) empty functional bilosomes, (**2**) curcumin and (**3**) methylene blue dissolved in THF:water (1:1) and (**4**) double-loaded functional bilosomes. The colors of the lines (**C**) correspond with the colors of the samples.

We applied the optimized bilosomes surface-modified with a triblock copolymer (system 7 in Table 1) to encapsulate curcumin (a hydrophobic drug, CUR) and methylene blue (a hydrophilic photosensitizer, MB) as a model hybrid cargo for potential anticancer treatment. During the anticancer research conducted by Khorsandi et al. [42], the synergistic effect of these compounds, applied in non-encapsulated (native) form was confirmed, which may be the result of ion pair formation. The authors showed increased cellular uptake of the ion pairing complex by human breast MDA-MB-231 cancer cells. For this reason, the simultaneous encapsulation of CUR with MB may lead to improved therapeutic efficiency, simultaneously enhancing biocompatibility of the hybrid cargo. The UV-Vis spectroscopy was applied to demonstrate the encapsulation of the hybrid cargo in the optimized polymeric bilosomes. Figure 6 shows the UV-Vis spectrum of the double-loaded bilosomes compared to the spectrum of the empty ones and the spectrum of CUR and MB dissolved in THF:water (1:1) mixture. The last three samples were used as control groups. The presence of representative peaks at 426 nm for CUR and at about 666 nm for MB provided evidence of effective encapsulation of both drugs in the proposed nanosystem. Generally, double encapsulation did not significantly change the formulation polydispersity. As expected, the loaded bilosomes showed a larger size compared to blank nanostructures. The mean particle size increased from 96.5 nm to 121.3 nm, which was probably caused by the two incorporated active agents into the nanocarrier structure.

The literature data reported that MB is widely used as a photosensitizer in a variety of applications, such as antibacterial and cancer treatments [43,44]. The high quantum yield of singlet oxygen generation and low toxicity of methylene blue makes it a promising candidate for photodynamic therapy (PDT), which is based on the systemic or local application of a photosensitive compound. The photocytotoxic reactions during PDT occur only within the morbid site (i.e., cancer cells), thereby allowing selective destruction of pathological tissues (without compromising healthy cells) [45]. Significantly, the clinical application of MB is limited due to its propensity for rapid chemical alteration in the biological environment [46]. Moreover, MB can occur as a monomeric or dimeric form in the aqueous solution. The active form of MB is monomeric, whilst the dimeric assemblies decrease its efficacy of PDT [42]. The active form of MB can be protected from environmental or enzymatic degradation after encapsulation into bilosomes. Thus, the preservation of the photoactive form of this photosensitizing agent may lead to increase the therapeutic efficiency of MB.

Importantly, in Figure 6 we observe a characteristic peak for the monomer form of MB, since dimers have maximum absorbance at about 590 nm [47]. According to Boccalini et al. [44], the increase in the absorbance of the monomer form of MB and disappearance of the absorption band of the dimer form may suggest that methylene blue was confined in nanostructures. The encapsulation efficiency achieved for curcumin and methylene blue was 70% and 85%, respectively. Higher entrapment efficiency of MB compared to CUR results from the fact that the encapsulation efficiency of hydrophilic drugs does not exhibit a strong dependence on the drug-to-lipid ratio. On the other hand, the EE of hydrophobic drugs depends on the properties of the acyl chains of nanocarriers, such as length or packing density. It is also connected with the changes in the drug-to-lipid ratio, which lead to reduced encapsulation efficiency of these compounds in the phospholipid bilayer [48]. For this reason, encapsulation of hydrophilic methylene blue was conducive to better entrapment of this molecule when compared with hydrophobic curcumin. These data showed that the simultaneous encapsulation of lipophilic and hydrophilic drugs indicates potential of the surface-modified bilosomes in further biomedical experiments.

## 4. Conclusions

We presented a significant report on a newly designed vesicular-type of functional phosphatidylcholine/cholesterol-based nanosystem whose surface was modified by biocompatible structural agents i.e., a bile salt—sodium cholate hydrate and a triblock copolymer—Pluronic P123. Three preparation methods—thin-film hydration, sonication and extrusion in combination with a homogenization technique were used to formulate the bilosomes, resulting in the new class of colloidal lipid-polymeric nanomaterials. The optimized vehicles indicated the nanometric size (D_H_ < 150 nm) at low polydispersity (PDI < 0.3) and negative surface charge (ζ ~ −35 mV), even after 28 days of the sample’s storage time. The kinetic stability of the selected bilosomes was additionally demonstrated by backscattering profiles evaluation. Finally, the high encapsulation efficiency of two model hybrid compounds, i.e., hydrophilic methylene blue and hydrophobic curcumin proved that a promising delivery platform for further biological studies was achieved.

Our further work will focus on in vitro evaluation of the co-loaded cargo release profiles in different pH and temperature conditions to check the biological potential of our nanocarriers in anticancer treatment, including intravenous and topical administration. Furthermore, the profound biological response of normal and cancer cells to the hybrid cargo, including biocompatibility, anticancer activity, internalization and bioimaging studies should be investigated. 

## Figures and Tables

**Table 1:** Composition of modified liposomes.

System	PC ^1^	Chol ^2^	Pluronic P123	SC ^3^	Water
	Contents/wt %				
1	1.00	0.30	-	-	98.70
2	1.00	0.30	0.60	-	98.10
3	1.00	0.30	-	0.25	98.45
4	1.00	0.30	-	0.50	98.20
5	1.00	0.30	-	1.00	97.70
6	1.00	0.30	0.60	0.25	97.85
7	1.00	0.30	0.60	0.50	97.60
8	1.00	0.30	0.60	1.00	97.10

^1^ PC: Phosphatidylcholine; ^2^ Chol: Cholesterol; ^3^ SC: Sodium Cholate.

## Supplementary Materials

The following are available online at https://www.mdpi.com/2079-4991/10/12/2472/s1, Figure S1: Influence of the modified liposomes preparation method on the (A) mean particle size (D_H_) and (B) polydispersity index (PdI). Figure S2: Influence of the storage time on (A) mean particle size (D_H_), (B) zeta potential (ζ) and (C) polydispersity index (PdI) for all samples after sonication. Figure S3: Time-dependent measurements of hydrodynamic diameter (D_H_) for the bilosomes incubated in the standard culture medium supplemented with 10% fetal bovine serum and antibiotics. Table S1: Composition of phospholipid vesicles.
