# Supplementary material for: Novel Surface-Modified Bilosomes as Functional and Biocompatible Nanocarriers of Hybrid Compounds
Source: Nanomaterials (Basel). 2020 Dec 10;10(12):2472. doi: 10.3390/nano10122472 (PMC7763575; doi:10.3390/nano10122472)
Supplement: Supplementary file 1 [file nanomaterials-10-02472-s001.pdf]

nanomaterials

Article

Novel Surface-Modified Bilosomes as Functional and Biocompatible Nanocarriers of Hybrid Compounds

Ewelina Waglewska, Agata Pucek-Kaczmarek * and Urszula Bazylińska *

Department of Physical and Quantum Chemistry, Faculty of Chemistry, Wroclaw University of Science and Technology, Wybrzeze Wyspianskiego 27, 50-370 Wroclaw, Poland; ewelina.waglewska@pwr.edu.pl

* Correspondence: agata.pucek@pwr.edu.pl (A.P.-K.); urszula.bazylinska@pwr.edu.pl (U.B.); Tel.: +48-71-3202183 (A.P.-K.; U.B.)

Table 1. Composition of phospholipid vesicles.

System	PC[a]	Chol[b]	Pluronic P123	Water
	Contents / wt %			
1	1.00	0.30	-	98.70
2	1.00	0.30	0.30	98.40
3	1.00	0.30	0.60	98.10
4	1.00	0.30	1.20	97.50

[a] PC: Phosphatidylcholine. [b] Chol: Cholesterol.

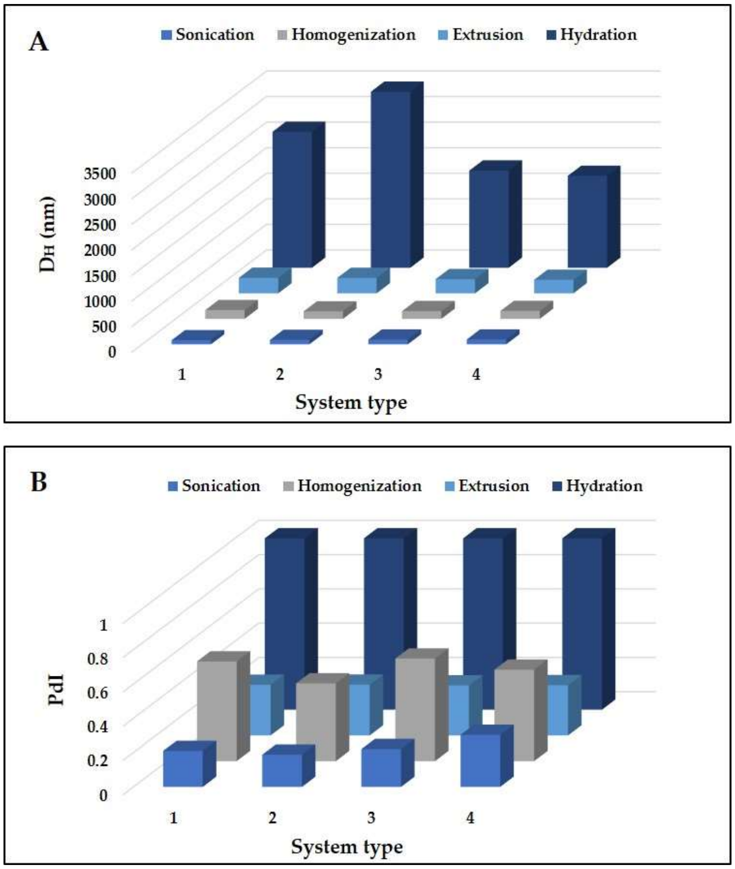

Figure S1. Influence of the modified liposomes preparation method on the (**A**) mean particle size (D_H) and (**B**) polydispersity index (PdI).

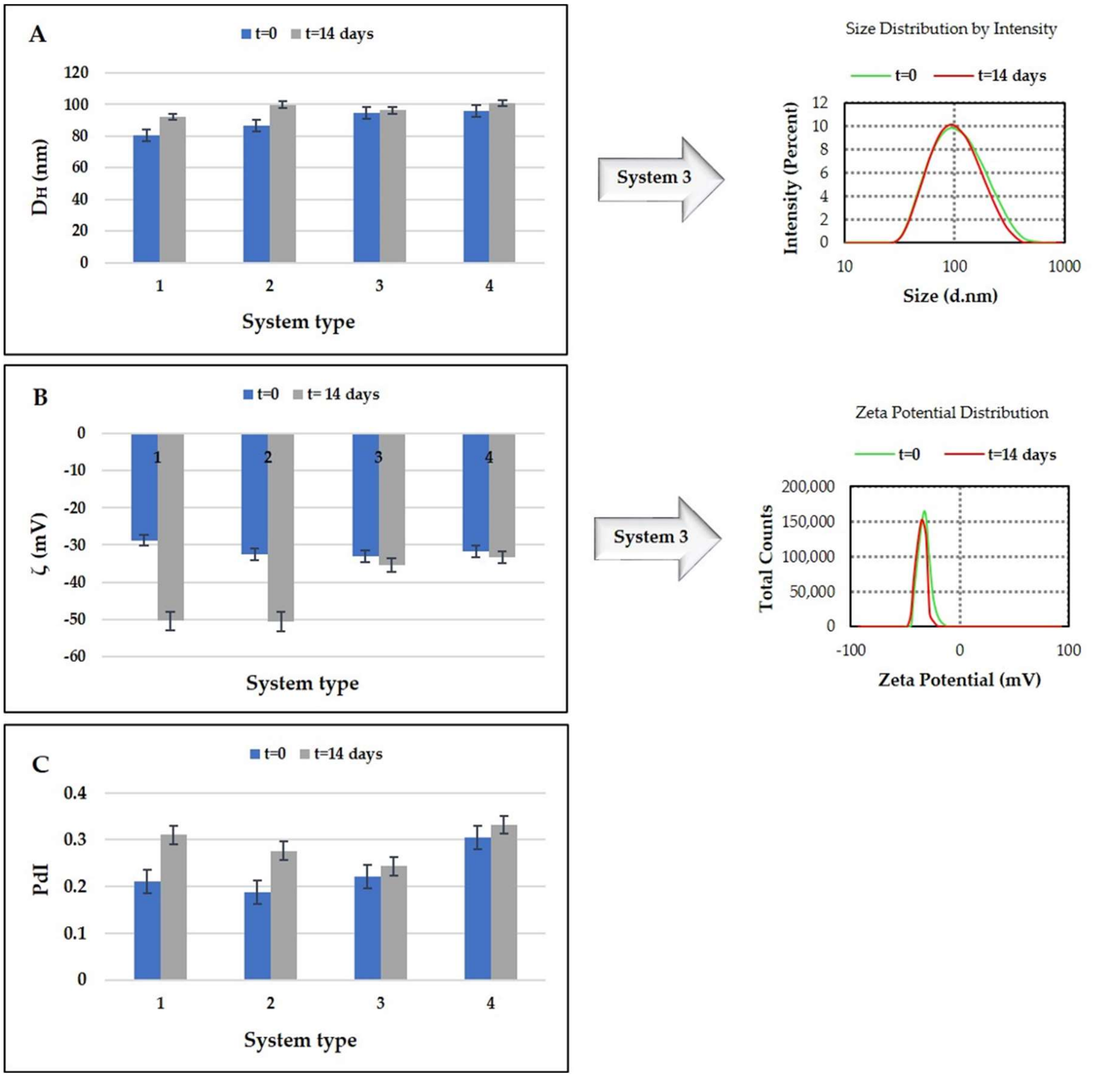

Figure S2. Influence of the storage time on (**A**) mean particle size (D_H), (**B**) zeta potential (ζ) and (**C**) polydispersity index (PdI) for all samples after sonication. .

Biological stability

Experiments concerning the measurements of biological stability against the nanocarrier agglomeration were provided by means of time-dependent D_H measurements (DLS) of the optimized bilosomes (system 7 from Table 1) in cell standard biological culture medium according to our previous investigations [Bazylińska & Saczko. *Nanoemulsion-templated polylelectrolyte multifunctional nanocapsules for DNA entrapment and bioimaging*. Colloids Surf B Biointerfaces 2016, 137, 191-202]. The study was conducted in Dulbecco's Modified Eagle's Medium, DMEM (Sigma) supplemented with 10% fetal bovine serum (Biowhittaker) and antibiotics (penicillin/streptomycin, Sigma) at 37°C, in 5% CO_2 humidified atmosphere. We applied a really high volume of the nanocarrier suspension in relation to the culture medium (1:4) to check its influence on the system stability process and the Pluronic stealth effect. Measurements of the hydrodynamic diameter were provided in eight different time intervals (from 15 min to 24 hours). The obtained results were presented in Fig. S3.

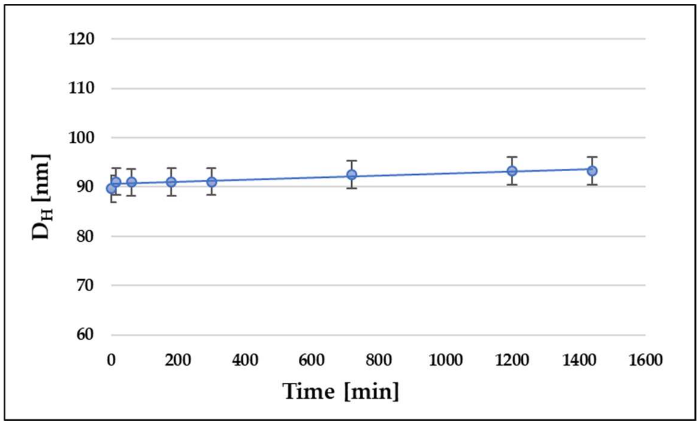

Figure S3. Time-dependent measurements of hydrodynamic diameter (D_H) for the bilosomes incubated in the standard culture medium supplemented with 10% fetal bovine serum and antibiotics.

© 2020 by the authors. Licensee MDPI, Basel, Switzerland. This article is an open access article distributed under the terms and conditions of the Creative Commons Attribution (CC BY) license (http://creativecommons.org/licenses/by/4.0/).
